# Crystal structure of 8-bromo-4-oxo-4*H*-chromene-3-carbaldehyde

**DOI:** 10.1107/S2056989015013250

**Published:** 2015-07-15

**Authors:** Yoshinobu Ishikawa

**Affiliations:** a School of Pharmaceutical Sciences, University of Shizuoka, 52-1 Yada, Suruga-ku, Shizuoka 422-8526, Japan

**Keywords:** crystal structure, chromone, hydrogen bonding, halogen bonding, π–π stacking

## Abstract

In the title compound, C_10_H_5_BrO_3_, a brominated 3-formyl­chromone, all atoms are essentially coplanar (r.m.s. = 0.0104 Å for the non-H atoms), with the largest deviation from the least-squares plane [0.028 (5) Å] being for one of the benzene C atoms. In the crystal, mol­ecules are linked through C—H⋯O hydrogen bonds, which are further assembled by face-to-face π–π stacking inter­actions [centroid–centroid distance between the pyran rings = 3.854 (4) Å]. Shorter contacts than the sum of van der Waals radii are observed between the Br and formyl O atoms [Br⋯O = 3.046 (4) Å, C—Br⋯O = 175.23 (18)° and Br⋯O—C = 132.6 (3)°], features that do indicate halogen bonding.

## Related literature   

For related structures, see: Ishikawa (2014*a* ▸,*b* ▸). For halogen bonding, see: Auffinger *et al.* (2004 ▸); Metrangolo *et al.* (2005 ▸); Wilcken *et al.* (2013 ▸); Sirimulla *et al.* (2013 ▸); Persch *et al.* (2015 ▸); Metrangolo & Resnati (2014 ▸); Mukherjee & Desiraju (2014 ▸).
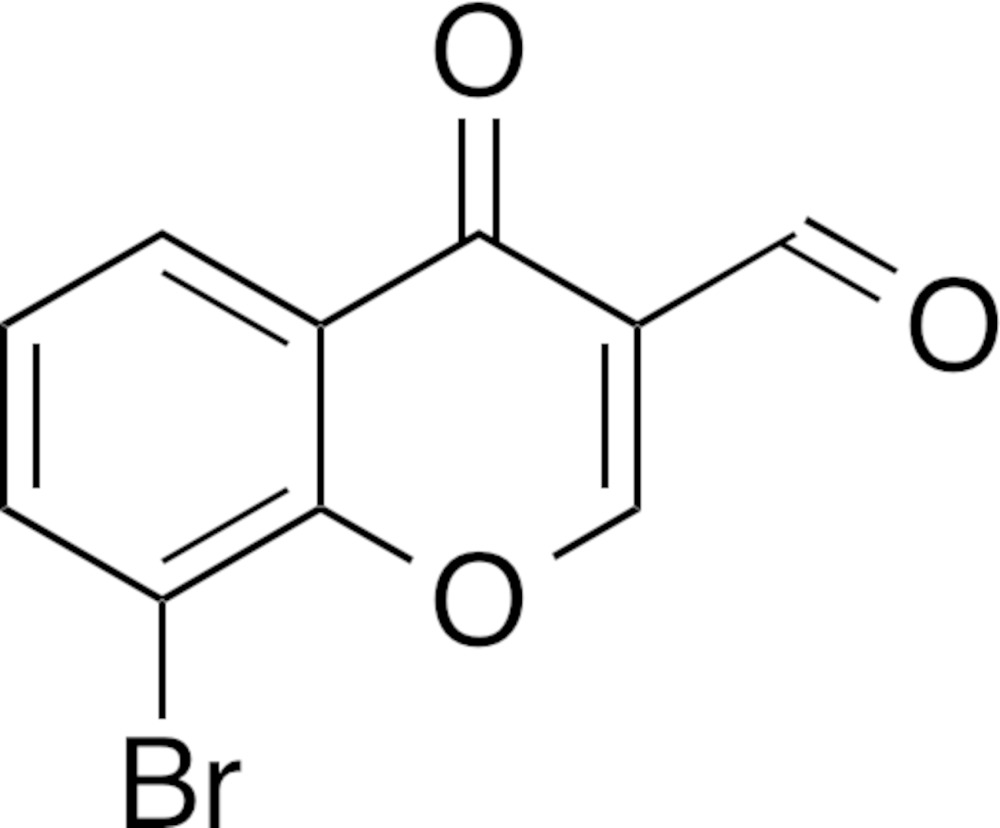


## Experimental   

### Crystal data  

C_10_H_5_BrO_3_
*M* *_r_* = 253.05
Monoclinic, 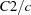

*a* = 27.908 (14) Å
*b* = 3.854 (3) Å
*c* = 19.145 (10) Å
β = 123.75 (4)°
*V* = 1712.1 (18) Å^3^
*Z* = 8
Mo *K*α radiation
μ = 4.79 mm^−1^
*T* = 100 K
0.37 × 0.10 × 0.07 mm

### Data collection  

Rigaku AFC-7R diffractometer
Absorption correction: ψ scan (North *et al.*, 1968 ▸) *T*_min_ = 0.546, *T*_max_ = 0.715
2556 measured reflections
1940 independent reflections
1280 reflections with *F* ^2^ > 2.0σ(*F* ^2^)
*R*_int_ = 0.020
3 standard reflections every 150 reflections intensity decay: −0.8%

### Refinement  

*R*[*F* ^2^ > 2σ(*F* ^2^)] = 0.037
*wR*(*F* ^2^) = 0.102
*S* = 1.02
1940 reflections
127 parameters
H-atom parameters constrained
Δρ_max_ = 1.40 e Å^−3^
Δρ_min_ = −1.27 e Å^−3^

### 

Data collection: *WinAFC Diffractometer Control Software* (Rigaku, 1999 ▸); cell refinement: *WinAFC Diffractometer Control Software*; data reduction: *WinAFC Diffractometer Control Software*; program(s) used to solve structure: *SIR92* (Altomare *et al.*, 1994 ▸); program(s) used to refine structure: *SHELXL97* (Sheldrick, 2008 ▸); molecular graphics: *CrystalStructure* (Rigaku, 2010 ▸); software used to prepare material for publication: *CrystalStructure*.

## Supplementary Material

Crystal structure: contains datablock(s) General, I. DOI: 10.1107/S2056989015013250/zl2634sup1.cif

Structure factors: contains datablock(s) I. DOI: 10.1107/S2056989015013250/zl2634Isup2.hkl

**Fig. 1.**
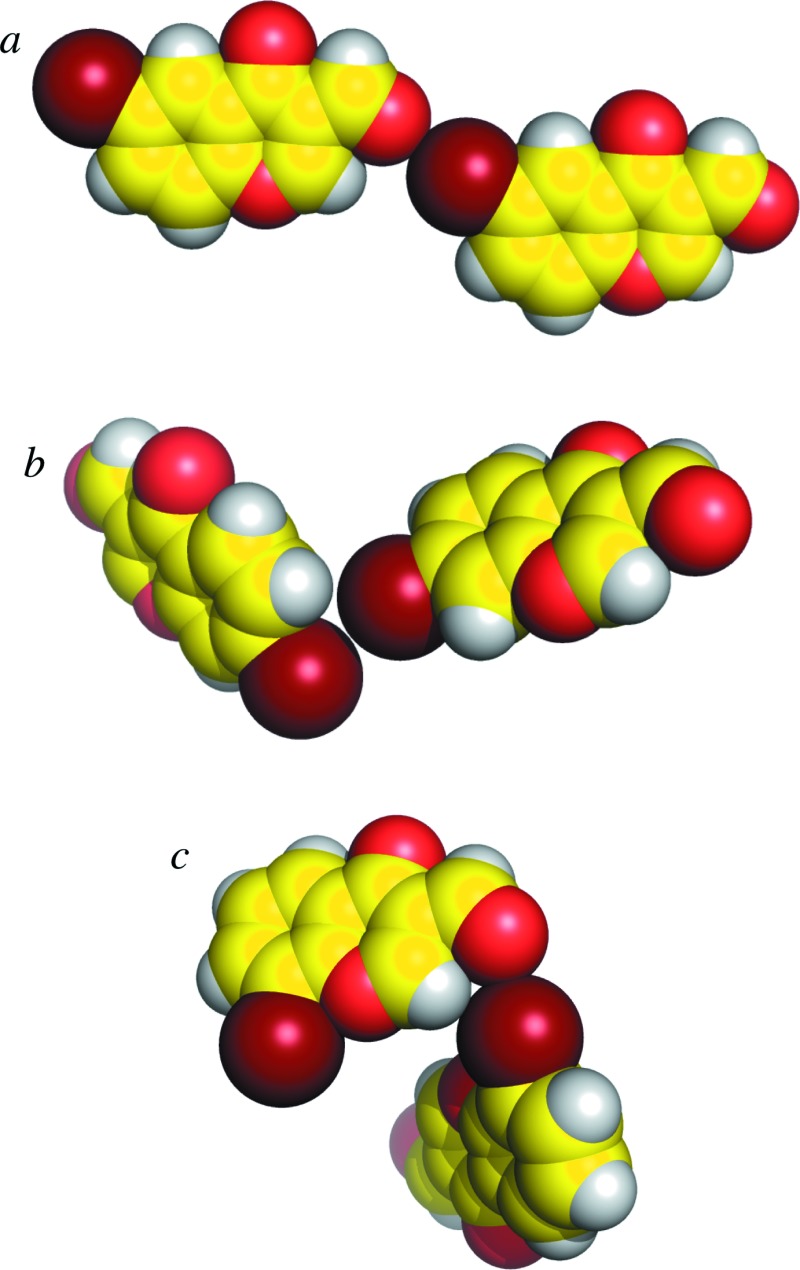
Sphere models of the crystal structures of (a) 6-bromo-4-oxo-4H-chromene-3-carbaldehyde (Ishikawa, 2014a), (b) 7-bromo-4-oxo-4H-chromene-3-carbaldehyde (Ishikawa, 2014b) and (c) the title compound (this work).

Supporting information file. DOI: 10.1107/S2056989015013250/zl2634Isup3.cml

a H a b H b c . DOI: 10.1107/S2056989015013250/zl2634fig1.tif
Sphere models of the crystal structures of (*a*) 6-bromo-4-oxo-4*H*-chromene-3-carbaldehyde (Ishikawa, 2014*a*), (*b*) 7-bromo-4-oxo-4*H*-chromene-3-carbaldehyde (Ishikawa, 2014*b*) and (*c*) the title compound (this work).

**Fig. 2.**
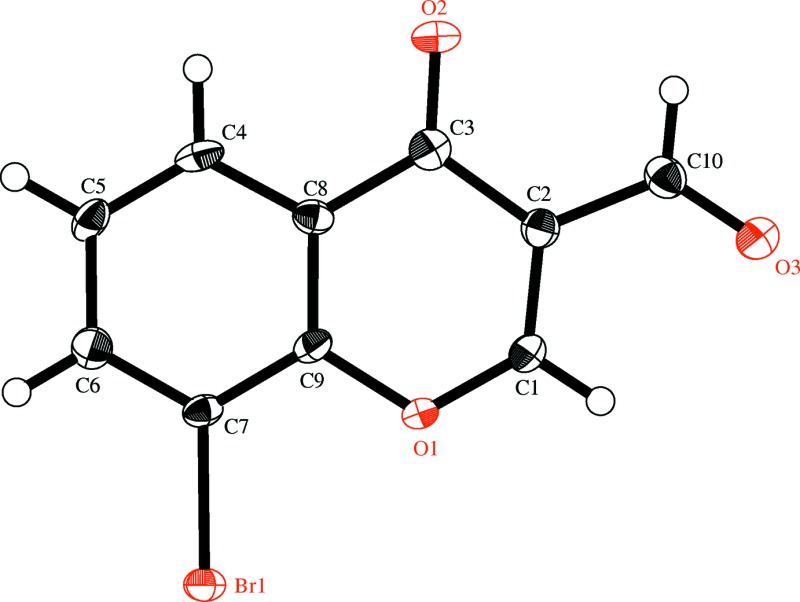
The molecular structure of the title compound with displacement ellipsoids drawn at the 50% probability level. Hydrogen atoms are shown as small spheres of arbitrary radius.

. DOI: 10.1107/S2056989015013250/zl2634fig2.tif
The mol­ecular structure of the title compound with displacement ellipsoids drawn at the 50% probability level. Hydrogen atoms are shown as small spheres of arbitrary radius.

**Fig. 3.**
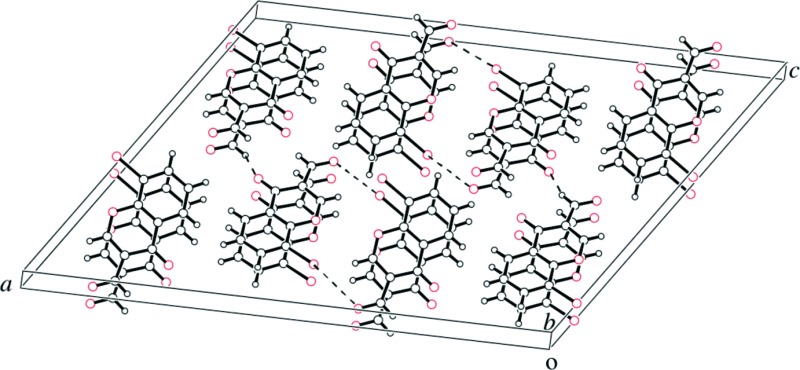
A packing view of the title compound. C—H···O hydrogen bonds and Br···O halogen bonds are represented by dashed lines.

. DOI: 10.1107/S2056989015013250/zl2634fig3.tif
A packing view of the title compound. C—H⋯O hydrogen bonds and Br⋯O halogen bonds are represented by dashed lines.

CCDC reference: 1412014

Additional supporting information:  crystallographic information; 3D view; checkCIF report

## Figures and Tables

**Table 1:** Hydrogen-bond geometry (, )

*D*H*A*	*D*H	H*A*	*D* *A*	*D*H*A*
C10H5O2^i^	0.95	2.54	3.375(5)	147(1)
C7Br1O3^ii^	1.89(1)	3.05(1)	4.934(6)	175(1)
C10O3Br1^iii^	1.21(1)	3.05(1)	3.962(6)	133(1)

Symmetry codes: (i) 
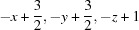
; (ii) 
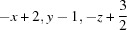
; (iii) 
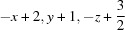
.

## supplementary crystallographic information

### S1. Comment

Halogen bonding has attracted much attention in medicinal chemistry, chemical biology, supramolecular chemistry and crystal engineering (Auffinger *et al.*, 2004, Metrangolo *et al.*, 2005, Wilcken *et al.*, 2013, Sirimulla *et al.*, 2013, Metrangolo & Resnati, 2014, Mukherjee & Desiraju, 2014, Persch *et al.*, 2015). I have recently reported the crystal structures of monobrominated 3-formylchromones 6-bromo-4-oxo-4*H*-chromene-3-carbaldehyde (Ishikawa, 2014*a*) and 7-bromo-4-oxo-4*H*-chromene-3-carbaldehyde (Ishikawa, 2014*b*). Halogen bonding is observed between the formyl oxygen atom and the bromine atom at 6-position in 6-bromo-4-oxo-4*H*-chromene-3-carbaldehyde (Fig. 1*a*). On the other hand, a type II halogen···halogen contact (Metrangolo & Resnati, 2014, Mukherjee & Desiraju, 2014) is found between the bromine atoms at 7-position in 7-bromo-4-oxo-4*H*-chromene-3-carbaldehyde (Fig. 1*b*). As part of my investigation into these types of chemical bonding, I herein report the crystal structure of the monobrominated 3-formylchromone 8-bromo-4-oxo-4*H*-chromene-3-carbaldehyde. The objective of this study is to reveal whether short contacts are observed for the bromine atom at 8-position in the solid state.

The mean deviation of the least-square planes for the non-hydrogen atoms is 0.0104 Å, and the largest deviation is 0.028 (5) Å for the C6 atom. These mean that these atoms are essentially coplanar (Fig. 2). In the crystal, the molecules are linked through C–H···O hydrogen bonds between the inversion-symmetry equivalents^i^ [i: –*x* + 3/2, –*y* + 3/2, –*z* + 1/2], which are further assembled by face-to-face π-π stacking interactions [centroid–centroid^ii^ distance between the pyran rings of the 4*H*-chromene units = 3.854 (4) Å, ii: *x*, *y* + 1, *z*], as shown in Fig. 3. Shorter contacts than the sum of van der Waals radii are observed between the bromine atoms at 8-position and the formyl O atoms [Br1···O3^iii^ = 3.046 (4) Å, C7–Br1···O3^iii^ = 175.23 (18)°, Br1···O3^iii^–C10^iii^ = 132.6 (3)°, iii: –*x* + 2, *y* – 1, –*z* + 3/2, Fig. 1*c*], features that indicate halogen bonding.

### S2. Experimental

To a solution of 3-bromo-2-hydroxyacetophenone (11.3 mmol) in *N*,*N*-dimethylformamide (20 ml) was added dropwise POCl_3_ (28.3 mmol) at 0 °C. After the mixture was stirred for 15 h at room temperature, water (50 ml) was added. The precipitates were collected, washed with water and dried *in vacuo* (yield: 55%). ^1^H NMR (400 MHz, CDCl_3_): *δ* = 7.40 (t, 1H, *J* = 7.8 Hz), 7.99 (dd, 1H, *J* = 1.4 and 7.8 Hz), 8.26 (dd, 1H, *J* = 1.4 and 8.3 Hz), 8.62 (s, 1H), 10.38 (s, 1H). Single crystals suitable for X-ray diffraction were obtained from a 1,2-dimethoxyethane solution of the title compound at room temperature.

### S3. Refinement

The C(*sp*^2^)-bound hydrogen atoms were placed in geometrical positions [C–H 0.95 Å, *U*_iso_(H) = 1.2*U*_eq_(C)], and refined using a riding model.

### Crystal data

**Table:**

C_10_H_5_BrO_3_	*F*(000) = 992.00
*M**_r_* = 253.05	*D*_x_ = 1.963 Mg m^−^^3^
Monoclinic, *C*2/*c*	Mo *K*α radiation, λ = 0.71069 Å
Hall symbol: -C 2yc	Cell parameters from 25 reflections
*a* = 27.908 (14) Å	θ = 15.0–17.3°
*b* = 3.854 (3) Å	µ = 4.79 mm^−^^1^
*c* = 19.145 (10) Å	*T* = 100 K
β = 123.75 (4)°	Plate, yellow
*V* = 1712.1 (18) Å^3^	0.37 × 0.10 × 0.07 mm
*Z* = 8	

### Data collection

**Table:**

Rigaku AFC-7R diffractometer	*R*_int_ = 0.020
ω scans	θ_max_ = 27.5°
Absorption correction: ψ scan (North *et al.*, 1968)	*h* = −20→36
*T*_min_ = 0.546, *T*_max_ = 0.715	*k* = −4→2
2556 measured reflections	*l* = −24→20
1940 independent reflections	3 standard reflections every 150 reflections
1280 reflections with *F*^2^ > 2.0σ(*F*^2^)	intensity decay: −0.8%

### Refinement

**Table:**

Refinement on *F*^2^	Secondary atom site location: difference Fourier map
*R*[*F*^2^ > 2σ(*F*^2^)] = 0.037	Hydrogen site location: inferred from neighbouring sites
*wR*(*F*^2^) = 0.102	H-atom parameters constrained
*S* = 1.01	*w* = 1/[σ^2^(*F*_o_^2^) + (0.0578*P*)^2^ + 0.244*P*] where *P* = (*F*_o_^2^ + 2*F*_c_^2^)/3
1940 reflections	(Δ/σ)_max_ = 0.004
127 parameters	Δρ_max_ = 1.40 e Å^−^^3^
0 restraints	Δρ_min_ = −1.27 e Å^−^^3^
Primary atom site location: structure-invariant direct methods	

### Special details

**Table:**

Refinement. Refinement was performed using all reflections. The weighted *R*-factor (*wR*) and goodness of fit (*S*) are based on *F*^2^. *R*-factor (gt) are based on *F*. The threshold expression of *F*^2^ > 2.0 *σ*(*F*^2^) is used only for calculating *R*-factor (gt).

### Fractional atomic coordinates and isotropic or equivalent isotropic displacement parameters (Å^2^)

**Table:**

	*x*	*y*	*z*	*U*_iso_*/*U*_eq_	
Br1	1.005108 (15)	−0.24742 (12)	0.90755 (2)	0.01865 (14)	
O1	0.93556 (11)	0.0942 (8)	0.73881 (17)	0.0180 (6)	
O2	0.76802 (12)	0.4314 (9)	0.60186 (18)	0.0261 (8)	
O3	0.86856 (12)	0.5554 (9)	0.50725 (18)	0.0277 (8)	
C1	0.91204 (17)	0.2414 (12)	0.6633 (3)	0.0188 (8)	
C2	0.85673 (18)	0.3611 (12)	0.6140 (3)	0.0188 (9)	
C3	0.81785 (19)	0.3273 (11)	0.6431 (3)	0.0199 (10)	
C4	0.81361 (17)	0.1171 (13)	0.7636 (3)	0.0220 (10)	
C5	0.83890 (17)	−0.0311 (12)	0.8415 (3)	0.0201 (10)	
C6	0.89618 (18)	−0.1386 (13)	0.8849 (3)	0.0212 (10)	
C7	0.92747 (16)	−0.0988 (12)	0.8491 (3)	0.0161 (9)	
C8	0.84441 (18)	0.1649 (11)	0.7261 (3)	0.0171 (10)	
C9	0.90209 (16)	0.0568 (11)	0.7709 (3)	0.0165 (9)	
C10	0.83814 (19)	0.5220 (12)	0.5332 (3)	0.0216 (10)	
H1	0.9357	0.2646	0.6422	0.0226*	
H2	0.7745	0.1891	0.7345	0.0264*	
H3	0.8174	−0.0606	0.8659	0.0241*	
H4	0.9138	−0.2392	0.9391	0.0254*	
H5	0.7997	0.6051	0.4991	0.0260*	

### Atomic displacement parameters (Å^2^)

**Table:**

	*U*^11^	*U*^22^	*U*^33^	*U*^12^	*U*^13^	*U*^23^
Br1	0.0141 (2)	0.0192 (3)	0.0204 (2)	0.0019 (2)	0.00822 (16)	0.0016 (2)
O1	0.0123 (13)	0.0238 (16)	0.0188 (14)	0.0028 (13)	0.0093 (12)	0.0037 (14)
O2	0.0140 (15)	0.034 (2)	0.0266 (17)	0.0055 (15)	0.0090 (13)	0.0042 (16)
O3	0.0218 (16)	0.039 (3)	0.0243 (16)	0.0025 (16)	0.0139 (14)	0.0031 (16)
C1	0.0189 (19)	0.022 (3)	0.0196 (18)	0.002 (3)	0.0131 (16)	0.000 (3)
C2	0.019 (2)	0.020 (3)	0.017 (2)	0.0013 (18)	0.0104 (18)	−0.0010 (17)
C3	0.021 (2)	0.017 (3)	0.022 (2)	−0.0013 (16)	0.0120 (18)	−0.0011 (16)
C4	0.0107 (19)	0.027 (3)	0.027 (3)	−0.0006 (19)	0.0094 (18)	−0.003 (2)
C5	0.018 (2)	0.024 (3)	0.023 (2)	−0.0074 (19)	0.0137 (18)	−0.0038 (19)
C6	0.020 (2)	0.024 (3)	0.018 (2)	−0.0038 (19)	0.0097 (18)	−0.0027 (18)
C7	0.0126 (19)	0.0132 (19)	0.022 (2)	−0.0012 (18)	0.0095 (17)	0.0004 (18)
C8	0.0148 (19)	0.015 (3)	0.018 (2)	−0.0004 (16)	0.0071 (17)	−0.0004 (15)
C9	0.0137 (19)	0.020 (3)	0.018 (2)	−0.0004 (18)	0.0102 (17)	−0.0031 (18)
C10	0.024 (3)	0.021 (3)	0.020 (2)	0.0023 (19)	0.0115 (18)	0.0006 (19)

### Geometric parameters (Å, º)

**Table:**

Br1—C7	1.892 (4)	C4—C8	1.405 (9)
O1—C1	1.337 (5)	C5—C6	1.393 (6)
O1—C9	1.381 (7)	C6—C7	1.387 (9)
O2—C3	1.224 (6)	C7—C9	1.387 (6)
O3—C10	1.205 (8)	C8—C9	1.402 (6)
C1—C2	1.367 (6)	C1—H1	0.950
C2—C3	1.475 (9)	C4—H2	0.950
C2—C10	1.469 (7)	C5—H3	0.950
C3—C8	1.469 (7)	C6—H4	0.950
C4—C5	1.370 (7)	C10—H5	0.950
			
Br1···O1	2.995 (4)	Br1···H1^i^	2.9874
O1···C3	2.875 (6)	Br1···H1^ii^	3.0464
O2···C1	3.581 (7)	Br1···H4^xii^	3.2048
O2···C4	2.881 (6)	Br1···H4^xiii^	3.1419
O2···C10	2.922 (8)	O1···H1^ii^	3.0722
O3···C1	2.799 (6)	O2···H3^xiv^	2.7748
C1···C7	3.585 (8)	O2···H5^v^	2.7432
C1···C8	2.760 (9)	O2···H5^vi^	2.5376
C2···C9	2.787 (7)	O3···H1^iv^	3.5037
C4···C7	2.773 (6)	O3···H3^viii^	2.9507
C5···C9	2.775 (9)	O3···H3^ix^	2.9772
C6···C8	2.796 (7)	O3···H4^viii^	2.5712
Br1···O3^i^	3.046 (4)	O3···H4^ix^	3.4743
O1···O1^ii^	3.378 (6)	C2···H5^iii^	3.4598
O1···C1^iii^	3.503 (6)	C3···H5^v^	3.3207
O1···C2^iii^	3.562 (6)	C4···H2^xv^	2.9781
O2···C8^iv^	3.544 (6)	C4···H2^xiv^	3.3174
O2···C10^v^	3.163 (5)	C4···H3^xiv^	3.3139
O2···C10^vi^	3.375 (5)	C5···H2^iii^	3.5171
O3···Br1^vii^	3.046 (4)	C5···H2^xv^	2.8601
O3···C5^viii^	3.452 (6)	C5···H4^iv^	3.5832
O3···C5^ix^	3.347 (6)	C6···H4^iv^	3.5732
O3···C6^viii^	3.266 (8)	C10···H3^viii^	3.4200
C1···O1^iv^	3.503 (6)	C10···H3^ix^	3.5839
C1···C10^iii^	3.527 (6)	H1···Br1^ii^	3.0464
C2···O1^iv^	3.562 (6)	H1···Br1^vii^	2.9874
C2···C10^iii^	3.497 (7)	H1···O1^ii^	3.0722
C3···C8^iv^	3.491 (7)	H1···O3^iii^	3.5037
C4···C5^iv^	3.511 (7)	H1···H4^viii^	3.5860
C5···O3^x^	3.452 (6)	H2···C4^xv^	3.3174
C5···O3^xi^	3.347 (6)	H2···C4^xiv^	2.9781
C5···C4^iii^	3.511 (7)	H2···C5^iv^	3.5171
C6···O3^x^	3.266 (8)	H2···C5^xiv^	2.8601
C7···C8^iii^	3.592 (6)	H2···H2^xv^	2.6156
C7···C9^iii^	3.486 (7)	H2···H2^xiv^	2.6156
C8···O2^iii^	3.544 (6)	H2···H3^iv^	3.5746
C8···C3^iii^	3.491 (7)	H2···H3^xiv^	2.3921
C8···C7^iv^	3.592 (6)	H3···O2^xv^	2.7748
C9···C7^iv^	3.486 (7)	H3···O3^x^	2.9507
C10···O2^v^	3.163 (5)	H3···O3^xi^	2.9772
C10···O2^vi^	3.375 (5)	H3···C4^xv^	3.3139
C10···C1^iv^	3.527 (6)	H3···C10^x^	3.4200
C10···C2^iv^	3.497 (7)	H3···C10^xi^	3.5839
Br1···H4	2.9274	H3···H2^iii^	3.5746
O2···H2	2.6153	H3···H2^xv^	2.3921
O2···H5	2.6505	H3···H5^x^	3.5442
O3···H1	2.4620	H3···H5^xi^	3.3525
C1···H5	3.2749	H4···Br1^xii^	3.2048
C3···H1	3.3068	H4···Br1^xiii^	3.1419
C3···H2	2.6753	H4···O3^x^	2.5712
C3···H5	2.7288	H4···O3^xi^	3.4743
C4···H4	3.2489	H4···C5^iii^	3.5832
C6···H2	3.2487	H4···C6^iii^	3.5732
C7···H3	3.2654	H4···H1^x^	3.5860
C8···H3	3.2750	H5···O2^v^	2.7432
C9···H1	3.1865	H5···O2^vi^	2.5376
C9···H2	3.2660	H5···C2^iv^	3.4598
C9···H4	3.2581	H5···C3^v^	3.3207
C10···H1	2.5371	H5···H3^viii^	3.5442
H1···H5	3.4755	H5···H3^ix^	3.3525
H2···H3	2.3112	H5···H5^vi^	3.0057
H3···H4	2.3430		
			
C1—O1—C9	118.5 (4)	C4—C8—C9	118.0 (4)
O1—C1—C2	125.2 (6)	O1—C9—C7	117.3 (4)
C1—C2—C3	119.7 (5)	O1—C9—C8	121.9 (4)
C1—C2—C10	118.2 (6)	C7—C9—C8	120.8 (5)
C3—C2—C10	122.1 (4)	O3—C10—C2	124.2 (4)
O2—C3—C2	122.5 (5)	O1—C1—H1	117.386
O2—C3—C8	123.2 (6)	C2—C1—H1	117.377
C2—C3—C8	114.3 (4)	C5—C4—H2	119.373
C5—C4—C8	121.3 (4)	C8—C4—H2	119.362
C4—C5—C6	120.0 (6)	C4—C5—H3	120.002
C5—C6—C7	120.1 (5)	C6—C5—H3	120.002
Br1—C7—C6	120.2 (3)	C5—C6—H4	119.976
Br1—C7—C9	120.0 (4)	C7—C6—H4	119.964
C6—C7—C9	119.8 (4)	O3—C10—H5	117.902
C3—C8—C4	121.6 (4)	C2—C10—H5	117.903
C3—C8—C9	120.4 (6)		
			
C1—O1—C9—C7	179.6 (4)	C8—C4—C5—C6	−0.2 (7)
C1—O1—C9—C8	0.7 (6)	C8—C4—C5—H3	179.8
C9—O1—C1—C2	0.1 (6)	H2—C4—C5—C6	179.8
C9—O1—C1—H1	−179.9	H2—C4—C5—H3	−0.2
O1—C1—C2—C3	−0.9 (7)	H2—C4—C8—C3	0.7
O1—C1—C2—C10	178.9 (4)	H2—C4—C8—C9	179.8
H1—C1—C2—C3	179.1	C4—C5—C6—C7	−0.6 (7)
H1—C1—C2—C10	−1.1	C4—C5—C6—H4	179.4
C1—C2—C3—O2	179.6 (4)	H3—C5—C6—C7	179.4
C1—C2—C3—C8	0.7 (6)	H3—C5—C6—H4	−0.6
C1—C2—C10—O3	0.1 (7)	C5—C6—C7—Br1	−179.6 (4)
C1—C2—C10—H5	−179.9	C5—C6—C7—C9	1.8 (7)
C3—C2—C10—O3	179.8 (4)	H4—C6—C7—Br1	0.4
C3—C2—C10—H5	−0.2	H4—C6—C7—C9	−178.2
C10—C2—C3—O2	−0.1 (7)	Br1—C7—C9—O1	0.4 (6)
C10—C2—C3—C8	−179.0 (4)	Br1—C7—C9—C8	179.2 (3)
O2—C3—C8—C4	0.3 (6)	C6—C7—C9—O1	179.1 (4)
O2—C3—C8—C9	−178.8 (4)	C6—C7—C9—C8	−2.1 (7)
C2—C3—C8—C4	179.1 (4)	C3—C8—C9—O1	−0.8 (6)
C2—C3—C8—C9	0.1 (6)	C3—C8—C9—C7	−179.6 (4)
C5—C4—C8—C3	−179.3 (4)	C4—C8—C9—O1	−179.9 (4)
C5—C4—C8—C9	−0.2 (7)	C4—C8—C9—C7	1.3 (6)

Symmetry codes: (i) −*x*+2, *y*−1, −*z*+3/2; (ii) −*x*+2, *y*, −*z*+3/2; (iii) *x*, *y*−1, *z*; (iv) *x*, *y*+1, *z*; (v) −*x*+3/2, −*y*+1/2, −*z*+1; (vi) −*x*+3/2, −*y*+3/2, −*z*+1; (vii) −*x*+2, *y*+1, −*z*+3/2; (viii) *x*, −*y*, *z*−1/2; (ix) *x*, −*y*+1, *z*−1/2; (x) *x*, −*y*, *z*+1/2; (xi) *x*, −*y*+1, *z*+1/2; (xii) −*x*+2, −*y*−1, −*z*+2; (xiii) −*x*+2, −*y*, −*z*+2; (xiv) −*x*+3/2, *y*+1/2, −*z*+3/2; (xv) −*x*+3/2, *y*−1/2, −*z*+3/2.

### Hydrogen-bond geometry (Å, º)

**Table:**

*D*—H···*A*	*D*—H	H···*A*	*D*···*A*	*D*—H···*A*
C10—H5···O2^vi^	0.95	2.54	3.375 (5)	147 (1)
C7—Br1···O3^i^	1.89 (1)	3.05 (1)	4.934 (6)	175 (1)
C10—O3···Br1^vii^	1.21 (1)	3.05 (1)	3.962 (6)	133 (1)

Symmetry codes: (i) −*x*+2, *y*−1, −*z*+3/2; (vi) −*x*+3/2, −*y*+3/2, −*z*+1; (vii) −*x*+2, *y*+1, −*z*+3/2.
