# Supplementary figures and images for: Crystal structure of 8-bromo-4-oxo-4H-chromene-3-carbaldehyde
Source: Acta Crystallogr E Crystallogr Commun. 2015 Jul 15;71(Pt 8):o572–3. doi: 10.1107/S2056989015013250 (PMC4571403; doi:10.1107/S2056989015013250)

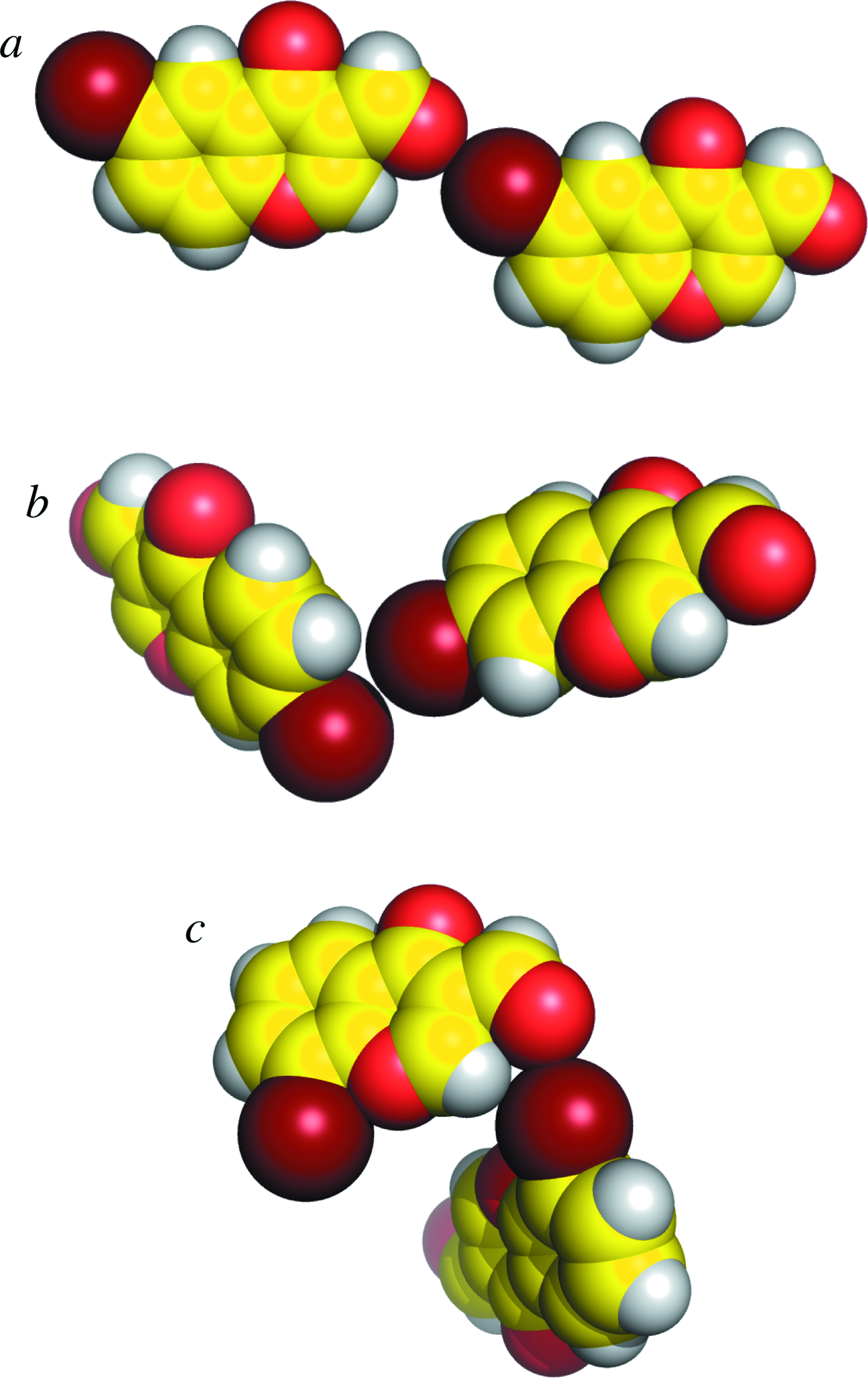

Supplement: Supplementary file 4 [file e-71-0o572-fig1.tif]

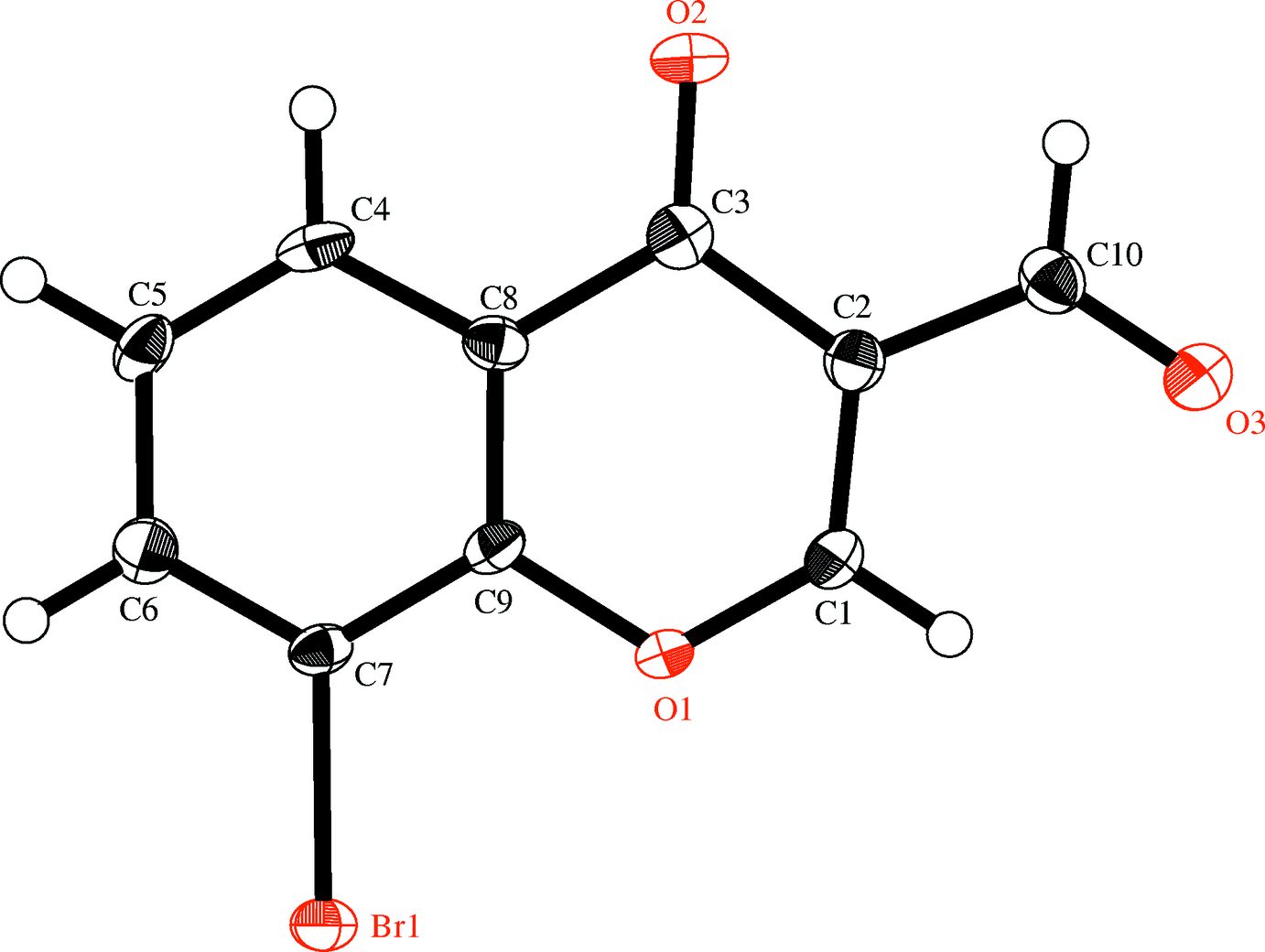

Supplement: Supplementary file 5 [file e-71-0o572-fig2.tif]

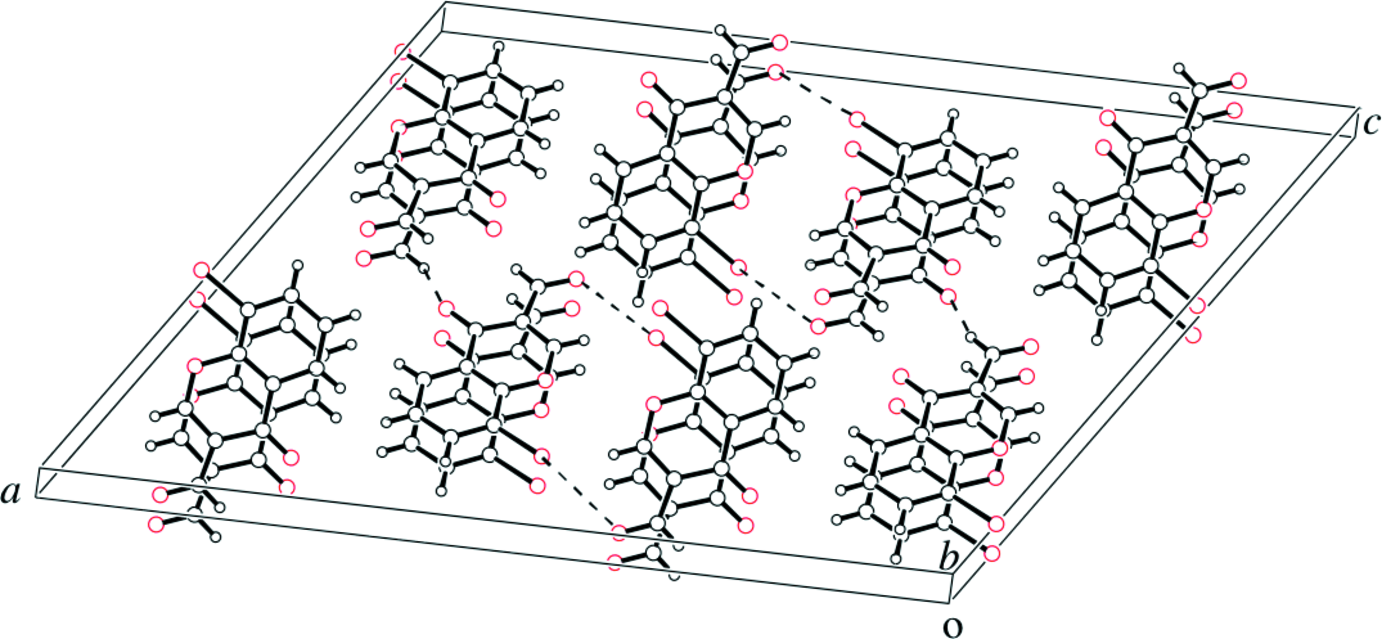

Supplement: Supplementary file 6 [file e-71-0o572-fig3.tif]
